# Professional Identity Formation in the Transition From Undergraduate to Postgraduate Medical Education: A Scoping Review

**DOI:** 10.1111/tct.70502

**Published:** 2026-09-02

**Authors:** Juliana Sá, Ana L. Da Silva, Pia Strand, Simon Kitto

**Affiliations:** ^1^ Faculty of Medicine, Health and Life Science Swansea University Swansea UK; ^2^ Department of Medical Sciences University of Aveiro Aveiro Portugal; ^3^ Faculty of Medicine Centre for Teaching and Learning Lund University Lund Sweden; ^4^ Institute of Learning Mohammed Bin Rashid University of Medicine and Health Sciences, Dubai Health Dubai UAE

## Abstract

**Background:**

The transition from undergraduate to postgraduate medical education is a crucial phase of professional development, requiring trainees to assume greater clinical responsibility and adapt to new social norms. This transition influences professional identity formation (PIF), which is important for safe practice, well‐being and resilience. We conducted a scoping review to map studies of PIF during this transition and synthesise the key findings of this body of literature.

**Methods:**

We searched five databases for studies published between January 2000 and December 2023. Eligibility criteria guided inclusion. We extracted relevant information and PRISMA‐ScR reporting elements and used inductive thematic analysis to synthesise findings.

**Results:**

Twenty‐one studies met the inclusion criteria, representing mixed‐methods, qualitative and quantitative designs and diverse theoretical approaches to PIF. Only one study explicitly defined ‘transition’. Most studies (*n* = 14) were descriptive; two were justification studies, and five aimed to clarify mechanisms, contextual influences and theoretical underpinnings. Four themes described factors influencing PIF in the transition from undergraduate to postgraduate education: navigating the self, the system, clinical practice and collaboration.

**Conclusions:**

Supporting early‐career doctors requires more than fostering resilience. Training environments must reduce systemic barriers, model professional values and create relational spaces where emerging doctors can belong, reflect and grow. Future research should move beyond description towards theoretically informed, multi‐context studies that examine mechanisms underlying these influences and generate transferable, actionable knowledge for those supporting trainees during this transition.

## Summary

- Trainees entering postgraduate training are engaged not merely in acquiring technical skills but in actively negotiating their identities.
- Creating protected spaces for structured reflection, including debriefing, supervision and peer‐learning activities, can directly support this process.
- Because systemic barriers, such as fragmented pathways, poor handover systems and unclear role expectations, operate independently of individual coping capacity, training environments should be actively audited for structural obstacles so that trainees' resilience is not redirected away from their development.
- Interprofessional and team‐based learning can be deliberately structured into training programmes, with supervisory relationships treated as formal developmental interventions rather than incidental arrangements.

## 1 Background

A transition in medical education has been defined as ‘a period of change in which medical students or medical doctors experience some form of discontinuity in their professional life space, forcing them to respond by developing new behaviours or changing their professional life space to cope with the new situation’ [1]. Among the many transitions in medical education, the move from undergraduate to postgraduate training is particularly significant. It marks the movement into the professional world, where training and work are combined, a context characterised by markedly different structures, levels of supervision and degrees of autonomy [2]. This transition is associated with increased responsibility for patient care [3] and the need to acquire social rules and norms to integrate into a community of practice [4]. These rules are often conveyed tacitly, significantly influencing trainees' professional identity and shaping their responses to conflicts (both internal and external), including doubt, adjustment and avoidance [5]. Literature describes transitions as liminal experiences that ‘significantly disrupt one's internal sense of self and place in a social system’ [6]. In this way, the transition from undergraduate to postgraduate medical education has been linked not only with heightened levels of stress and anxiety among trainees [7] but also with reported implications for clinical care and patient safety [8]. The transition carries risks for PIF that extend beyond the general challenges of medical training. Studies indicate that newly qualified doctors face a marked and abrupt shift in responsibility, moving from supervised learners to independent practitioners virtually overnight—a change described as the ‘intern shock’ or ‘reality shock’ [7, 8]. Consequently, understanding PIF, especially in the transition to postgraduate education [9], whether through direct entry into speciality training or through foundation/preparatory years, is essential for building and sustaining a workforce of competent and resilient physicians [10, 11]. In medical education, PIF has been defined as ‘a representation of self, achieved in stages over time during which the characteristics, values, and norms of the medical profession are internalised, resulting in an individual thinking, acting, and feeling like a physician’ [12]. PIF is recognised as essential for the well‐being [13, 14], resilience [15, 16] and retention [17] of medical students and practitioners. Recent efforts have been made to synthesise the extensive literature on PIF [18, 19]. However, these syntheses have examined PIF across the medical education continuum [18] or in relation to specific pedagogical approaches [19], whereas existing reviews of the transition to postgraduate training have foregrounded preparedness, competence and well‐being rather than identity. To date, no synthesis has focused specifically on PIF at this juncture. A PIF lens reframes the well‐documented difficulties of this transition, such as the loss of belonging, not as transient performance problems to be endured but as identity work that can be recognised and deliberately supported. These works describe potential conflicts between trainees' values and beliefs and their professional roles and expectations [20]. If this conflict is not addressed, it can undermine the trainees' sense of belonging or purpose, reduce self‐esteem or self‐efficacy and lead to distress or burnout. However, we suggest that if these defining experiences are recognised and appropriately supported, they may enhance tolerance for ambiguity, facilitate meaning‐making, promote socialisation into communities of practice and strengthen enculturation and commitment to the profession [20]. Cruess et al. emphasised that PIF must be explicitly supported through intentional curricular strategies [20]. Given the profound significance of this transition in formally assuming the role of a licensed physician, it necessitates a particularly comprehensive understanding of PIF that both supports trainees' development and safeguards patient safety [21]. Without explicit and targeted support for PIF at this moment, trainees may adopt maladaptive coping strategies—such as emotional distancing or suppression of uncertainty—that, if left unaddressed, can solidify into enduring patterns of practice inconsistent with safe and compassionate care [5, 20]. These consequences carry direct implications not only for individual well‐being but also for patient safety, given that errors and near‐misses are disproportionately reported in the early months following transition [8]. This makes the undergraduate‐to‐postgraduate transition a critical window for intervention and underscores the need for a dedicated synthesis of evidence on PIF during this process.

This scoping review aims to (i) map the extent and nature of the literature investigating PIF in the transition from undergraduate to postgraduate medical education and (ii) synthesise the key findings of this body of literature. These aims are operationalised in two research questions (Section 2.1), which the results and discussion address in turn.

## 2 Methods

This scoping review was conducted using the framework established by Arksey and O'Malley [22] and subsequently refined by Levac et al. [23]. It adhered to the reporting standards of PRISMA‐ScR (Preferred Reporting Items for Systematic Reviews and Meta‐Analyses extension for Scoping Reviews). The scoping review methodology was selected as particularly appropriate for this topic, given the breadth of the field, the diversity of study designs in the literature and the aim of mapping and synthesising existing evidence to address the review research questions.

### 2.1 Research Questions

The following research questions were addressed:

- RQ1: What are the main characteristics of studies investigating professional identity formation in the transition from undergraduate to postgraduate medical training?
- RQ2: What are the key findings of the literature on professional identity formation in the transition from undergraduate to postgraduate medical training?

### 2.2 Search Strategy

The search strategy, developed with a university health librarian, aimed to identify published literature through a two‐step process. Starting in March 2023, the initial search in Medline involved analysing the words in article titles, abstracts and index terms used to describe the articles. Term harvesting was conducted on six key papers. These articles were used to develop a comprehensive search strategy by identifying keywords in titles and abstracts and indexing terms for categorising relevant studies. These terms were tested against MeSH terms and included where appropriate. The initial search returned 238 papers, and the terms were deemed sufficient for the primary search strategy. The final set of search keywords incorporating Boolean operators and MeSH terms was defined based on the analysis (see Data S1). The publication period spanned from 1 January 2000 to 31 December 2023, representing an active research period on PIF.

### 2.3 Selection Criteria

The developed search strategy was then applied to the databases MEDLINE, Web of Science, Scopus, ASSIA and PsycINFO using the specified inclusion and exclusion criteria (Table 1).

**TABLE 1:** Study eligibility criteria.

Inclusion criteria	Topic: The content must be related to professional identity in the transition from undergraduate to postgraduate, including experiences and perceptions. All articles were required to explicitly address the concepts of ‘professional identity’ and ‘transition’ Methods: All types included (e.g., quantitative, qualitative and mixed methods) Publication type: Articles could be primary research, evaluation studies, reviews or editorials Language: Articles in English, Spanish and Portuguese were considered Year of publication: From 1 January 2000 to 31 December 2023
Exclusion criteria	Professional identity in other healthcare professions that does not include medicine Other transitions periods, different from undergraduate to postgraduate Papers not available after request to the author Books, chapters, white papers, dissertations or thesis

### 2.4 Mapping of Study Characteristics—Data Charting and Extraction

The first stage of the process involved manually screening titles and discarding those unrelated to the research questions while keeping a record of the excluded articles and the reasons for exclusion. Duplicate studies were removed, and the remaining abstracts were carefully examined. Full‐text articles were retrieved and closely reviewed in accordance with the inclusion and exclusion criteria. Mendeley's reference management system was used to facilitate the storage and organisation of records. In line with established scoping review methodology [24], relevant data from each study were systematically extracted using a standardised data charting form developed in Microsoft Excel beforehand. The data collected included information on study characteristics such as year, country and setting; research aims; study design; participant details; and data collection, analysis methods and results. Data on PIF during the transition were analysed and synthesised. The data charting process was iterative, with adjustments necessary to ensure a consistent and comprehensive approach across studies. Data charting sequence is described step by step in Table 2.

**TABLE 2:** Data charting sequence.

Step 1	The first author (J.S.) and second author (A.D.S.) independently reviewed the records (titles and abstracts), documenting their decisions on whether to include or exclude and provided explanations for their choice with Covidence. After the full‐text review was conducted, discrepancies were noted and discussed between the authors (J.S. and A.D.S.) until consensus was reached
Step 2	The authors as a team identified the data elements required to address the research questions and subsequently developed a coding manual and data extraction form, both implemented using a Microsoft Excel spreadsheet. The extraction form was drafted by J.S. and reviewed by A.D.S., in line with recommended procedures [25]
Step 3	The form was pretested by J.S. and A.D.S. to ensure inter‐coder reliability. They independently piloted the coding book and charting form on a sample of four randomly selected articles. Results were compared in an inter‐rater session, leading to the finalisation of the extraction form
Step 4	The final extraction form captured: title, author(s), publication year, country of origin, publication type, research approach, research question(s) addressed, theoretical framework used, study conclusions and directions for future research

### 2.5 Understanding PIF During the Transition—Thematic Analysis

Data on PIF during the transition were extracted and analysed using inductive thematic analysis, as outlined by Braun and Clarke [26]. The process began with familiarisation, during which J.S. and A.D.S. read and re‐read the extracted data to develop a thorough understanding of the content. Initial codes were generated through pattern coding to capture recurring features and ideas. These codes were then organised into subthemes that reflected underlying patterns across the dataset. J.S. and A.D.S. independently reviewed and compared the subthemes and coding structure, with all data undergoing a thorough review to ensure consistency and depth of analysis. Through discussion and reflection, a consensus was reached on a final thematic map that represented key themes. The thematic model was further discussed by the authors J.S., P.S., S.K. and A.D.S., leading to the identification of additional insights and the final articulation of four themes describing factors influencing PIF during the transition from undergraduate to postgraduate training (Table 5).

### 2.6 Ethical Considerations

This study is a scoping review of previously published literature and did not involve human participants or unpublished data; formal research ethics approval was therefore not required.

## 3 Results

**FIGURE 1:**
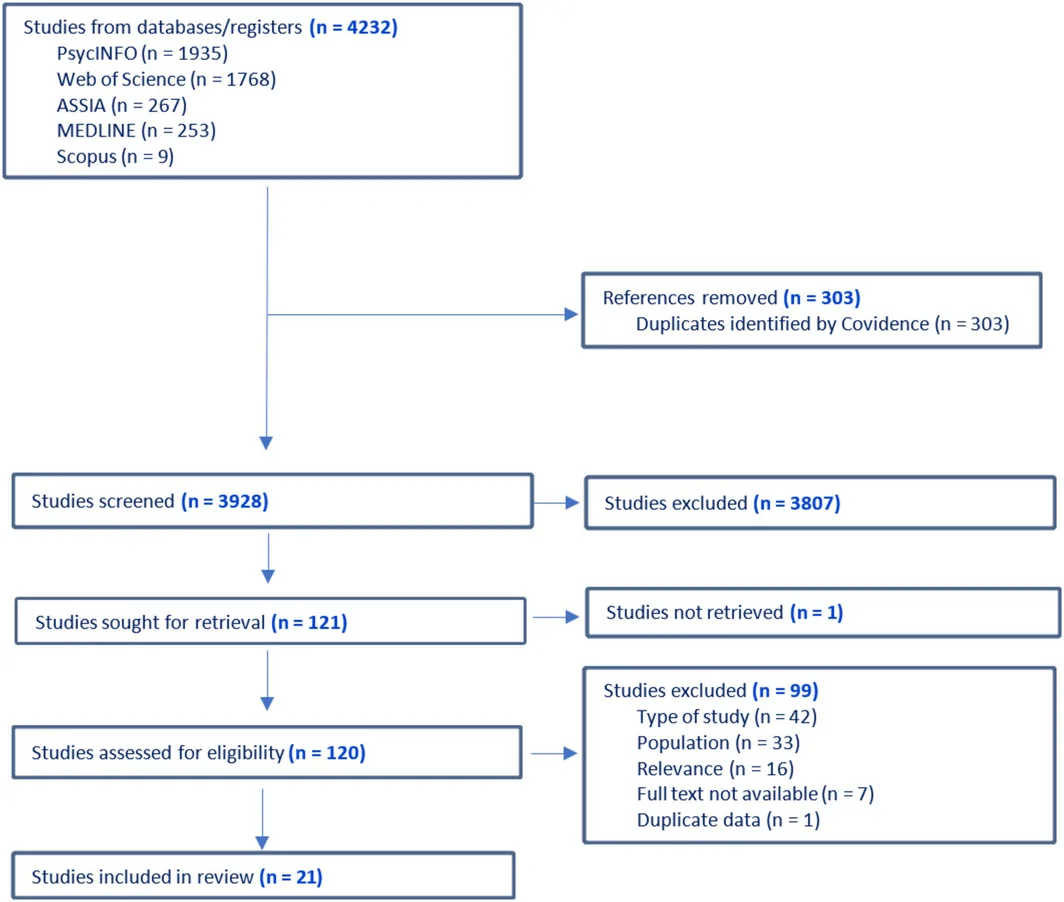
PRISMA flow diagram illustrating the study selection process.

Following the screening process, illustrated in the PRISMA flow diagram (Figure 1), 21 studies met the inclusion criteria for this review. A complete list of the included articles is provided in Data S1. The results are presented in two sections addressing the two research questions. Section 3.1 reports the characteristics of the included studies (RQ1); Section 3.2 reports the thematic analysis of findings on PIF during the transition between undergraduate and postgraduate medical education (RQ2).

### 3.1 Study Characteristics

First authors were affiliated with institutions in the United States (*n* = 6), the United Kingdom (*n* = 5), the Netherlands (*n* = 2), Iran (*n* = 1), Denmark (*n* = 1), Australia (*n* = 1), Taiwan (*n* = 1) and Germany (*n* = 1), with three additional studies reflecting cross‐national author teams or multi‐site data collection: the United Kingdom and Taiwan (*n* = 1), Brazil and Ireland (*n* = 1) and the Netherlands in collaboration with other countries (*n* = 1). The included studies were published between 2010 and 2023, with the majority (*n* = 11) published between 2020 and 2022. Most studies focused exclusively on postgraduate trainees. Three studies included both medical students and postgraduate trainees in their sampling [28, 39, 43]. The final dataset included qualitative studies (*n* = 18), of which nine were longitudinal and nine were not longitudinal, one quantitative longitudinal study (*n* = 1), one survey/quantitative longitudinal study (*n* = 1) and one mixed‐methods study (*n* = 1). In longitudinal studies, follow‐up periods ranged from 3 months [32] to 3 years [43]. Summary of the main characteristics of the studies are described in Table 3.

**TABLE 3:** Studies characteristics.

Authors	Year	Title	Country	Study design (defined by authors)	Transition phase	Internship/Foundation year	Refs.
Satterfield and Becerra	2010	Developmental Challenges, Stressors and Coping Strategies in Medical Residents: A Qualitative Analysis of Support Groups	USA	Qualitative (not longitudinal)	Both	No	[28]
Bearman et al.	2011	Participation and Progression: New Medical Graduates Entering Professional Practice	Australia	Qualitative (not longitudinal)	Postgraduate	No	[39]
Szymczak and Bosk	2012	Training for Efficiency: Work, Time and Systems‐Based Practice in Medical Residency	USA	Qualitative (not longitudinal)	Postgraduate	No	[34]
De Lasson et al.	2016	Professional Identity Formation in the Transition From Medical School to Working Life: A Qualitative Study of Group‐Coaching Courses for Junior Doctors	Denmark	Qualitative (not longitudinal)	Postgraduate	No	[44]
Jones et al.	2016	‘He's Going to Be a Doctor in August’: A Narrative Interview Study of Medical Students' and Their Educators' Experiences of Aligned and Misaligned Assistantships	UK	Quantitative longitudinal	Undergraduate	Yes	[40]
Wainwright et al.	2017	Coming Back From the Edge: A Qualitative Study of a Professional Support Unit for Junior Doctors	UK	Qualitative longitudinal	Postgraduate	Yes	[30]
van den Broek et al.	2017	Medical Students' Preparation for the Transition to Postgraduate Training Through Final Year Elective Rotations	Netherlands	Qualitative longitudinal	Undergraduate	Yes	[3]
Monrouxe et al.	2017	Association of Professional Identity, Gender, Team Understanding, Anxiety and Workplace Learning Alignment With Burnout in Junior Doctors: A Longitudinal Cohort Study	UK/Taiwan	Mixed methods	Postgraduate	Yes	[7]
Ahmadinia et al.	2019	Medical Residents' Viewpoint About the Effective Stressors on Professional Identity Formation During Residency in Tehran University of Medical Sciences: A Qualitative Study	Iran	Qualitative (not longitudinal)	Postgraduate	No	[41]
Yardley et al.	2020	‘What Do We Do, Doctor?’ Transitions of Identity and Responsibility: A Narrative Analysis	UK	Qualitative (not longitudinal)	Postgraduate	Yes	[29]
Brown et al.	2020	Becoming a Clinician: Trainee Identity Formation Within the General Practice Supervisory Relationship	UK	Qualitative longitudinal	Postgraduate	No	[33]
Chang et al.	2020	The Transition From Medical Student to Resident: A Qualitative Study of New Residents' Perspectives	USA	Qualitative longitudinal	Postgraduate	Yes	[31]
van den Broek et al.	2020	Social Identification With the Medical Profession in the Transition From Student to Practitioner	Netherlands	Survey/quantitative longitudinal	Both	No	[36]
Montagna et al.	2021	Transition to Clinical Practice During the COVID‐19 Pandemic: A Qualitative Study of Young Doctors' Experiences in Brazil and Ireland	Brazil/Ireland	Qualitative (not longitudinal)	Postgraduate	No	[25]
Chaou et al.	2021	The Evolution of Medical Students' Preparedness for Clinical Practice During the Transition of Graduation: A Longitudinal Study From the Undergraduate to Postgraduate Periods	Taiwan	Qualitative longitudinal	Postgraduate	No	[42]
Brown et al.	2021	A Phenomenological Study of New Doctors' Transition to Practice, Utilising Participant‐Voiced Poetry	UK	Qualitative longitudinal	Both	Yes	[43]
Wolff et al.	2021	Facilitated Transitions: Coaching to Improve the Medical School to Residency Continuum	USA	Qualitative longitudinal	Postgraduate	No	[38]
Santivasi et al.	2022	Reframing Professional Identity Through Navigating Tensions During Residency: A Qualitative Study	USA	Qualitative (not longitudinal)	Postgraduate	No	[35]
van Duin et al.	2022	Junior Doctors' Experiences With Interprofessional Collaboration: Wandering the Landscape	Netherlands/multi‐country	Qualitative longitudinal	Postgraduate	No	[27]
Zink et al.	2022	Hopes and Fears: A Qualitative Analysis of the Intern Perspective at the Start of EM Residency	USA	Qualitative (not longitudinal)	Postgraduate	No	[45]
Homberg et al.	2023	Experience‐Based Learning During the Final Year–Quantitative Content Analyses of Students' Self‐Reports	Germany	Qualitative longitudinal	Undergraduate	No	[32]

#### 3.1.1 Classification Category

Cook et al. [46] categorise study types into three types: description, justification and clarification. This classification provides a structured framework for understanding the purpose, design and contribution of educational research. Among the reviewed studies, most (*n* = 14) were classified as descriptive studies [3, 25, 27, 30, 31, 34, 35, 36, 38, 39, 42, 43, 44, 45]. These focused on outlining educational interventions or innovations without systematically evaluating outcomes. Two studies were justification studies reporting the results of intentionally designed educational activities and placement characteristics that could influence PIF during transition [7, 28]. One justification study reported on a post‐graduation group‐coaching course that supported PIF, adaptation to medical culture, career planning and the management of a healthy work–life balance [7]. The other justification study described a coaching programme aimed at encouraging reflection on successes and challenges experienced during medical school, demonstrating the feasibility and acceptability of this approach to facilitate transition [28]. Five studies aimed at clarification [29, 32, 33, 40, 41] by illuminating the mechanisms, contextual influences and theoretical underpinnings of PIF during transitional periods.

#### 3.1.2 Theoretical Frameworks Applied to Conceptualise PIF

Among the theoretical frameworks used to examine PIF, five studies (*n* = 5) explicitly stated that they applied a specific theoretical framework to introduce the concept of PIF as part of the study background. We classified a study as applying an explicit theoretical framework where the framework was stated up front and structured the study's design and analysis; studies that instead drew on an established perspective post hoc, to interpret findings rather than to initially design the study, were not counted in this figure, as detailed below. The studies, which are classified as clarification studies (Table 4), portray PIF as a dynamic, socially mediated process shaped by relationships, context and systemic conditions [29, 32, 33, 40, 41]. Brown et al. [33] applied the multiple and multidimensional transitions theory, proposed by Jindal‐Snape et al. [47] and subsequently adapted to the context of new doctors' transitions to practice [48], to explore the complexities of this professional passage. They demonstrate how identity develops through dialogue and negotiation between the trainee and the supervisor, although Santivasi et al. [35] describe it as a reciprocal interaction among the person, behaviour and environment. Two studies grounded their work in social identity theory [37, 39], which frames professional identity as a socially constructed phenomenon shaped through interpersonal interactions, experiential learning and clinical engagement, alongside the social identity approach, to examine how doctors develop and negotiate their professional identities. Van den Broek et al. [39] emphasise belonging and emotional connection as essential mechanisms of identification with the medical profession and portray identity as the central organising concept of the student‐to‐resident transition, evolving through interaction with patients and teams. Complementing these relational and developmental perspectives, Szymczak and Bosk [34] show how institutional norms of efficiency and time management influence the professional self. These clarifying studies highlight identity formation as an ongoing negotiation between individual meaning‐making and the social structures and contexts of medical training. Beyond these five, a further three studies drew on an existing conceptual perspective to interpret their findings rather than to structure the study's design from the outset and were therefore not counted among the five studies using an explicit theoretical framework. In two studies [28, 43], PIF was approached applying Cruess et al.'s [20] perspective, conceptualising PIF as a transformative process of professional socialisation. One study [42] adopted the concept of ‘landscape identity’, originally described by Reinders et al. [49] as an interprofessional identity that complements the professional physician identity, to investigate the interprofessional dimensions of identity formation. One out of the 21 studies provides an explicit, operational definition of ‘transition’ [29]. The remaining studies describe the transition in nominal terms without a shared understanding of its specific characteristics.

**TABLE 4:** Classification category [46].

Classification category	*N* = 21	Studies
Description	14	[3, 25, 27, 30, 31, 34, 35, 36, 38, 39, 42, 43, 44, 45]
Justification	2	[7, 28]
Clarification	5	[29, 32, 33, 40, 41]

### 3.2 Thematic Analysis of Findings on PIF During Transition

**FIGURE 2:**
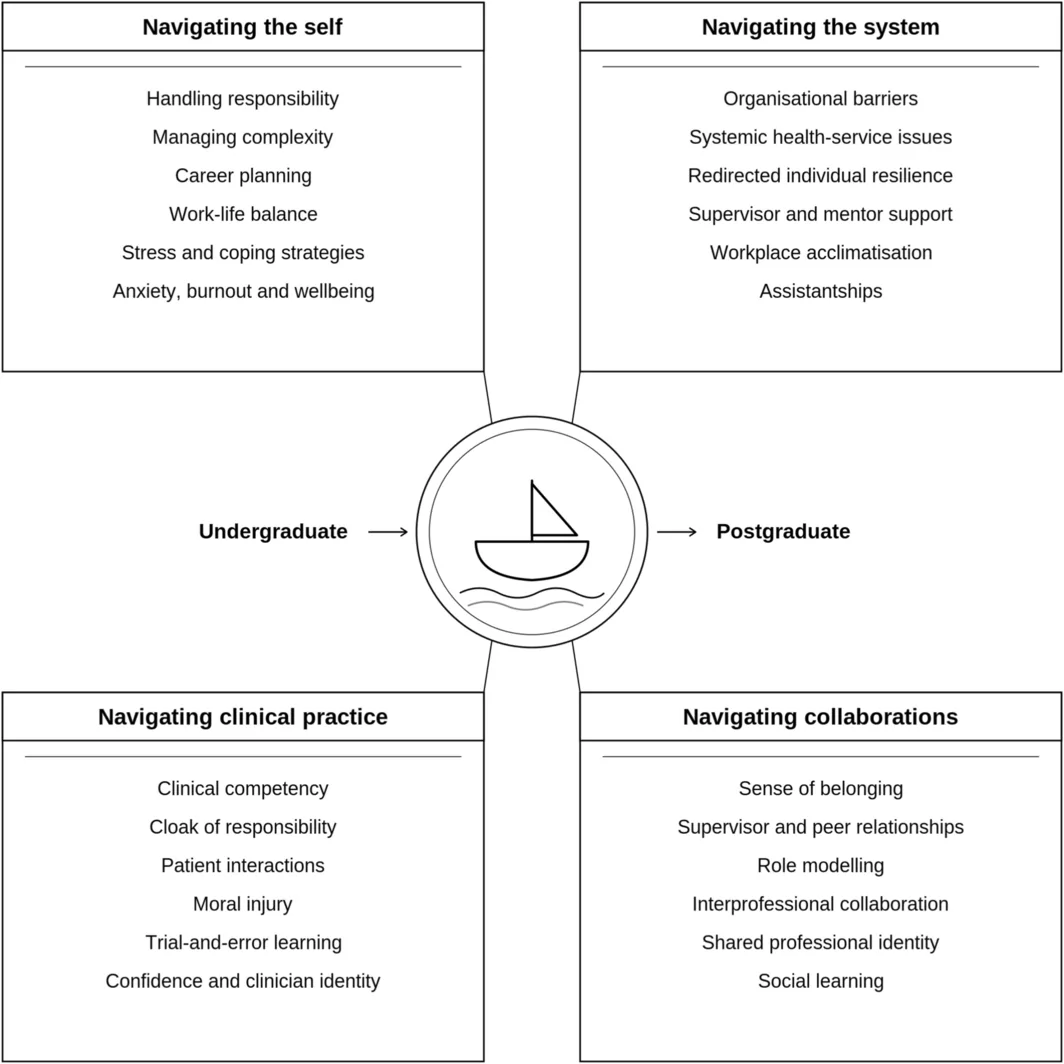
Themes identified in the literature that were associated with professional identity formation during the transition from undergraduate to postgraduate medical education.

Our thematic analysis generated four themes that describe key findings of the reviewed literature, pointing to critical factors influencing PIF during the transition from undergraduate to postgraduate education: navigating the self; navigating the system; navigating clinical practice; and navigating collaboration (Figure 2). Table 5 describes the studies from which each theme emerged and the number of studies related to each theme.

**TABLE 5:** Themes emerging in each study.

Themes illustrating factors influencing PIF	Number of studies related to each theme	Studies where the theme emerged
Navigating the self	12	[7, 27, 28, 29, 30, 31, 32, 33, 34, 35, 36, 37]
Navigating the system	4	[29, 33, 34, 38]
Navigating clinical practice	9	[27, 28, 29, 31, 36, 38, 39, 40, 41]
Navigating collaborations	9	[7, 26, 32, 35, 38, 39, 42, 43, 44]

#### 3.2.1 Navigating the Self

Several factors influencing PIF during the transition studied relate to individual learner traits and adaptive responses in medical training contexts. These include the capability to handle increasing responsibility [32, 33, 35], to tolerate and manage complexity [33], to engage in effective career planning [30] and to sustain work–life balance [30, 35]. Other factors, such as stress management [29, 37], utilisation of adaptive coping strategies [31, 36, 37], coping with anxiety and burnout [7, 34] and support for personal well‐being [27, 28], are recognised as vital to learners' identity formation and resilience.

#### 3.2.2 Navigating the System

The ‘system’ emerged as a theme linked to descriptions of organisational and systemic barriers that trainees had to overcome, thereby influencing their professional identity. The ‘system problems’ were described as obstacles to efficiency, with the note that ‘the system’ encompasses various organisational barriers [33]. Trainees described this system as an ‘entity that works against them’ [33] in their efforts to prioritise and anticipate tasks. Furthermore, trainees redirected their ‘individual resilience’ to address ‘systemic health‐service issues’ [29]. However, the ‘organisational support’ provided by supervisors or mentors was also identified as a crucial factor in facilitating PIF during the transition [38]. Trainees emphasised the importance of assistantships in easing workplace acclimatisation, noting that these opportunities reduced their anxieties about starting work [34].

*The ‘system’ emerged as a theme linked to descriptions of organisational and systemic barriers that trainees had to overcome, thereby influencing their professional identity*.

#### 3.2.3 Navigating Clinical Practice

Developing clinical competency emerged as a key factor influencing PIF during the transition from undergraduate to postgraduate medical education. This includes gaining an understanding of competence [29, 36] and adopting the ‘cloak of responsibility’ in clinical practice [40]. It further involves participating in patient interactions [27, 39], challenging personal beliefs, values and experiences of moral injury [29] and embracing one's identity and role as a clinician [27, 29, 36]. This acceptance of identity may also require disrupting or reframing the self [31, 41]. Identity formation primarily develops through experiential learning [27, 40], often through trial and error [40]. This learning focuses on clinical performance and involves building confidence [28].

#### 3.2.4 Navigating Collaborations

Collaboration is an influencing factor on PIF described as fostering a sense of belonging, strengthening supervisor–trainee relationships and facilitating exposure to effective role models [38, 39, 43]. Group interactions can serve as essential social learning opportunities, providing cues for behavioural adaptation that aid integration into new professional environments [44]. Through interactions within the team, individuals can form new connections [26, 35], which in turn reinforce a sense of belonging [7, 39]. Relationships with supervisors [32, 38, 39] and peers [39] are consistently recognised as vital to PIF during transitional phases. Furthermore, in the context of interprofessional collaboration, the literature highlights the value of engaging with diverse professional groups [35, 39, 42]. Such interactions promote constructive cross‐professional feedback and contribute to the development of a shared professional identity [42]. Within these interprofessional settings, both collective experiences of team‐based collaboration and the fostering of new interprofessional relationships have been independently linked to PIF [26, 43].

## 4 Discussion

The aim of this scoping review was to map the characteristics of studies examining PIF during the transition from undergraduate to postgraduate medical education and to synthesise the key findings of the literature in this area. In what follows, we first consider what the pattern of study characteristics reveals about the field's maturity (RQ1). We then discuss the key findings of the literature, namely, the four themes describing factors that influence PIF, and their implications for practice and research (RQ2).

### 4.1 Over‐Representation From the Global North

The study characteristics indicate an overrepresentation of studies from Anglo‐Saxon countries. Although first authors were affiliated with institutions across nine countries, 11 of the 21 studies originated from the United States (*n* = 6) or the United Kingdom (*n* = 5) and given the well‐established importance of contextual factors in shaping PIF [50], this concentration may limit the transferability of findings to other cultural, structural or healthcare contexts [51].

### 4.2 Predominantly Descriptive Studies

Our findings reveal that predominantly descriptive studies (*n* = 14) on PIF in the transition were more common, whereas justification (*n* = 2) and clarification studies (*n* = 5) were considerably less common. This distribution is not merely a bibliometric observation; it signals a field that has mapped the terrain of PIF during transition without yet interrogating the mechanisms or conditions that shape it. The resulting gap illustrates the need for future research to move beyond description towards theoretically informed, explanatory and evaluative inquiry, examining not only what occurs during this transition but also how and why particular experiences, relationships and structures either support or obstruct the formation of professional identity.

### 4.3 The Concept and Trajectory of Transition

The included studies consistently framed the transition from undergraduate to postgraduate education as an important phase for PIF [31, 36], although this importance was more often asserted than empirically examined. However, only one of the included studies offered an explicit, operational definition of ‘transition’ as a theorised construct [29]. Yardley et al. [29] characterised it as a multidimensional process involving psychological, social and educational adaptation. Twenty out of twenty‐one studies described the transition in nominal terms without any specific shared understanding of its characteristics. Without a shared understanding of when transition begins, what marks its end and what processes are constitutive of it, the conditions under which PIF is promoted or hindered remain ambiguous. This represents one of the most significant structural gaps in the current evidence base and reveals that the transition period has been consistently assumed rather than theorised across the literature. Furthermore, the review revealed a focus on only two distinct transitional trajectories within the included literature: seven studies examined the specific passage from medical school to a foundation or internship year prior to speciality training [3, 7, 29, 30, 31, 40, 43], whereas the remaining studies described the transition directly from medical school into speciality training. This structural heterogeneity, unremarked upon in the primary literature, compounds the definitional problem: Studies may be describing qualitatively different transitions under the same label, which further impedes synthesis and comparison. Together, these findings signal a major challenge to the design of any educational intervention to support PIF along the medical education/training continuum. The lack of clarity in the conceptualisation of transition is highly problematic. Theorising practice context is widely regarded as one of the key determinants of how to intervene in clinical practice [52], and by extension, understanding the learning context is crucial in the design of an effective curriculum that directly addresses PIF [53] and medical education more generally [54].

*This represents one of the most significant structural gaps in the current evidence base and reveals that the transition period has been consistently assumed rather than theorised across the literature*.

Four themes were identified in the literature related to transitioning from undergraduate to postgraduate training: navigating the self; navigating the system; navigating clinical practice; and navigating collaboration. The theme of navigating the self captures the intrapersonal domain of factors influencing PIF in the process of transition. Factors include managing increasing responsibility [32, 33, 35], tolerating complexity [33], career planning [30], stress management [29, 37], adaptive coping [31, 36, 37] and preventing anxiety and burnout [55, 56]. Learners are required to reconcile personal values with institutional expectations, a process that can simultaneously generate growth and conflict [29]. This tension is identified in 12 of the 21 included studies (Table 5), suggesting that the transition phase consistently places learners in a position of identity negotiation, aligning with prior research emphasising resilience, self‐regulation and adaptive coping as essential competencies during this phase [36, 37].

Another—navigating the system—emerged in four studies that explore how learners are caught in a structure/agency dilemma in which structural factors directly shape opportunities for learners to actively engage in PIF. Trainees in the included studies described the healthcare system as an entity that ‘works against them’ [33], thereby redirecting individual resilience towards managing systemic health‐service failures rather than personal development [29]. Organisational barriers to efficiency were experienced not as peripheral inconveniences but as constitutive features of the training environment [33], and assistantships and supervisory support were reported to ease workplace acclimatisation and reduce anxiety about entering practice [38, 55]. In this way, the low frequency with which systemic factors were examined, relative to the prominence with which trainees described their impact, points to a research gap. This disproportion suggests that the literature has systematically underweighted institutional and structural determinants of PIF relative to individual and relational ones, a pattern that risks individualising what are, in part, structural problems and implying that trainees bear primary responsibility for navigating environments that might not be designed to support their development.

The third theme—navigating clinical practice—identifies the breadth of coping strategies used by learners, such as developing an understanding of competence [29, 36], adopting the ‘cloak of responsibility’ [40], engaging in patient interactions [27, 39], confronting moral injury [29] and reframing professional self‐concept [31, 41]. PIF in this theme was predominantly understood as experiential and often trial and error in nature [27, 40], mediated through clinical performance [38] and the gradual construction of confidence [28]. The ‘cloak of responsibility’ functions simultaneously as a developmental catalyst and a source of burden, and its dual nature captures a tension that the descriptive literature has identified but that the clarification literature has only partially explained. Nine studies in total addressed this theme (Table 5). Of the five studies classified as clarification type (Table 4), three engaged directly with the mechanisms of clinical practice as an identity‐shaping context [29, 40, 41], but none examined how the scaffolding of clinical responsibility, including timing, pace or support structures, might be optimised to support PIF rather than simply to ensure clinical performance.

The fourth and final theme—navigating collaborations—which was identified in nine studies, highlights the relational and social dimensions of PIF. Both horizontal peer relationships and vertical supervisory relationships were reported to play complementary roles, consistent with the conceptualisation of PIF as co‐constructed within communities of practice [57]. The literature documents a range of collaborative contexts in which team‐based interactions occur that contain cues for behavioural adaptation and reinforce belonging [39, 49, 56], supervisor–trainee relationships that model professional values and provide developmental support [32, 38, 39], peer relationships that normalise shared experience [39] and interprofessional encounters that promote cross‐professional feedback and contribute to a shared professional identity [42]. Notably, although these 12 studies identified relational factors as influential, the mechanisms through which these relationships shaped identity were examined in only a subset of studies, and theoretical engagement with social identity or socio‐cultural frameworks was limited to two studies that explicitly applied social identity theory [37, 39]. This points to a further gap where the majority of included studies treated collaboration as a facilitating context without examining the specific interactional processes through which belonging, identity affirmation or professional socialisation occur.

### 4.4 Practical Implications for Transition Support

Our findings have practical implications for clinical teachers supporting early‐career doctors through this transition (Table 6). Despite the predominantly descriptive nature of the included literature, the convergence of evidence across themes is sufficient to offer interim, evidence‐informed guidance. Clinical teachers should recognise that trainees entering postgraduate training are engaged in active identity negotiation, not merely acquiring technical skills, and that creating protected spaces for structured reflection through debriefing and supervision directly supports this process. The studies examining systemic barriers suggest that clinical teachers and programme directors should actively audit training environments for structural obstacles, including fragmented pathways, poor handover systems and unclear role expectations. Because such barriers operate independently of individual coping capacity, addressing them at source prevents trainees' resilience from being consumed by compensating for system failures rather than invested in their development. Understanding clinical practice as the primary site of PIF, the sequencing and scaffolding of clinical responsibility matters: Clinical teachers should introduce the ‘cloak of responsibility’ progressively and with explicit reflective support, rather than treating full immersion as an unqualified developmental good. Studies addressing collaboration suggest that interprofessional and team‐based learning should be deliberately structured into training programmes, with supervisory relationships treated as formal developmental interventions rather than incidental arrangements. These are some preliminary guidelines that can be implemented by clinical tutors and supervisors; however, a robust, evidence‐informed framework, grounded in theoretically informed and evaluative research, needs to be developed to inform the practical approach to creating more supportive training environments that facilitate this transition.

**TABLE 6:** Translating the four themes into provisional, evidence‐informed implications for clinical teachers.

Theme	What the review found	Practical guidance for clinical teachers
Navigating the self	Trainees engage in active identity negotiation, reconciling personal values with professional expectations; stress, coping and burnout dominate this theme	Provide protected, structured reflection (debriefing, supervision, peer groups); normalise identity questions as an expected part of development rather than a sign of weakness
Navigating the system	Systemic barriers redirect trainees' resilience towards compensating for organisational failures rather than development	Audit training environments for fragmented pathways, poor handover and unclear role expectations; address structural obstacles rather than expecting individual adaptation
Navigating clinical practice	Identity develops experientially through graded clinical responsibility, often by trial and error	Sequence and scaffold clinical responsibility (e.g., assistantships); introduce the ‘cloak of responsibility’ progressively, with explicit reflective support
Navigating collaboration	Belonging, role models and supervisory, peer and interprofessional relationships shape identity	Deliberately structure interprofessional and team‐based learning; treat supervisory relationships as formal developmental interventions rather than incidental arrangements

### 4.5 PIF in the Transition: A Future Research Agenda

Despite the recognised importance of identifying influences on PIF [9], there remains limited clarity about how these influences operate in practice, particularly during transitions, which are widely recognised as periods of intense learning and identity development [10]. Only five of the 21 included studies explicitly grounded PIF in a theoretical framework, and only one offered an operational definition of the transition itself; theorisation of this phase is therefore not merely scarce but nearly absent. Although several studies reference organisational‐level factors [29, 33, 34, 38, 44], they do not provide a detailed analysis of organisational support during transitions or its specific impact on PIF. These gaps highlight the need for future research to adopt theoretically grounded, multi‐level approaches that critically examine how different contexts shape PIF during transitions.

The concept of ‘culture’ was frequently invoked in the results section but seldom explicitly defined. Culture is frequently discussed in relation to adverse outcomes such as moral injury [29], strained workplace relationships [30, 33] and suboptimal supervisory practices [31]. The influence of context and culture should be explicitly defined, analysed and addressed to support doctors meaningfully during their transition. Although existing studies recognise the influence of workplace relationships, supervisory dynamics and hidden curricula [39, 56], this scoping review highlights the need for a better understanding of the underlying mechanisms of PIF during the transition from undergraduate to postgraduate education. Exploring cultural and contextual factors through studies with a comprehensive theoretical framework is crucial for advancing this field. The development of professional identity is dependent on organisational contexts and has been described as a ‘fluid, dynamic, and socially constructed phenomenon’ [50]. Adopting a sociological perspective provides deeper insight into how PIF evolves and adapts to the complexities of healthcare settings. Comparative studies across different healthcare systems, specialities and training environments could also help determine whether certain influences are broadly applicable or context specific; this should include both instrumental and cross‐case analyses [58].

Finally, longitudinal studies with consistent follow‐up periods and clearly defined outcome measures will be vital to capturing the full complexity of PIF during this critical phase. Addressing these research gaps will deepen our understanding of the transition process. It could, in turn, inform policies and training programmes that promote patient care and safety while creating nurturing environments for emerging medical professionals. Additional research is necessary to establish a stronger theoretical foundation for understanding these dynamics that may foster more supportive environments for new medical professionals.

## 5 Limitations

Although an extensive search of bibliographic databases was conducted, some relevant articles might have been overlooked. Excluding papers involving other healthcare professions could result in missing data that might have been identified in research on related health education fields. Furthermore, most of the literature originates in the United States and the United Kingdom, which could influence the results, given the importance of context in the transition process. Additionally, the included studies were not subjected to quality assessment, in line with standard scoping review practices. Critics have noted that the lack of critical appraisal typical of scoping reviews may limit their ability to identify gaps due to poor‐quality research, as the papers were not evaluated for quality, contrary to standard methods.

## 6 Summary

This scoping review provides a critical overview of the literature on PIF during the transition from undergraduate to postgraduate medical education. This transition is widely recognised as an important moment in medical training, with significant implications for patient safety. However, our analysis reveals that it is frequently treated as a time‐bounded event rather than as an ongoing, socially embedded process. This perspective limits understanding of what is genuinely at stake during this phase of PIF within professional, organisational and cultural contexts. The literature reviewed is primarily descriptive and weighted towards individual coping strategies, adaptive responses and intrapersonal resilience. Although these dimensions are important, this emphasis risks obscuring the equally significant role of social structures, cultural norms and institutional frameworks in shaping PIF during transition. The four themes identified in this review collectively suggest that identity formation during this period is not a process trainees navigate alone, but one that is co‐produced through the systems they work within, the clinical experiences they encounter and the supervisory and collegial relationships they are afforded. Although the research base needs further exploration, the findings of this review are relevant to clinical teachers and those responsible for supporting early‐career doctors. Supporting trainees through this transition, therefore, demands a response that is simultaneously institutional, cultural and relational: reducing systemic barriers that redirect individual resilience away from development, modelling and affirming professional values and ensuring that mentorship, peer connection and interprofessional collaboration are treated as deliberate developmental provisions. Future research should build on this descriptive foundation with theoretically informed, multi‐context studies that examine the mechanisms underlying these influences and generate transferable, actionable knowledge for those supporting trainees at this transition.

## Conclusions

This review reveals a gap in research on professional identity formation during the undergraduate‐to‐postgraduate transition, which risks obscuring the significant role of social structures, cultural norms and institutional frameworks in shaping PIF. PIF is not an individual journey alone, but a socially embedded process shaped by clinical responsibility, institutional conditions and supervisory, peer and interprofessional relationships. Supporting early‐career doctors, therefore, demands training environments that deliberately reduce systemic barriers, model professional values and create relational spaces in which emerging doctors can belong, reflect and grow. Although theoretically grounded, multi‐context research on the mechanisms of PIF in this transition is still needed, clinical teachers need not wait for it: Graduated clinical responsibility, protected structured reflection, supervision treated as a developmental intervention and deliberately structured interprofessional teams are concrete, immediately available ways to support new doctors' emerging professional identities.

*Supporting early‐career doctors, therefore, demands training environments that deliberately reduce systemic barriers, model professional values and create relational spaces in which emerging doctors can belong, reflect and grow*.

## Author Contributions

**Pia Strand:** methodology, validation, supervision, writing – review and editing. **Simon Kitto:** validation, writing – review and editing, supervision, conceptualisation. **Ana L. Da Silva:** supervision, data curation, methodology, validation, writing – review and editing, conceptualisation, investigation. **Juliana Sá:** conceptualisation, investigation, writing – original draft, formal analysis.

## Funding

The authors have nothing to report.

## Conflicts of Interest

The authors declare no conflicts of interest.

## Supporting information


**Table S1:** Keywords used in the search.
**Table S2:** Condensed extraction table.

## Data Availability

The data that support the findings of this study are available on request from the corresponding author. The data are not publicly available due to privacy or ethical restrictions.
