# Supplementary material for: Professional Identity Formation in the Transition From Undergraduate to Postgraduate Medical Education: A Scoping Review
Source: Clin Teach. 2026 Sep 2;23(5):e70502. doi: 10.1111/tct.70502 (PMC13537030; doi:10.1111/tct.70502)
Supplement: Supplementary file 1 [file TCT-23-e70502-s001.docx]

**Supplement 1.**

**Table 1. Keywords used in the search**

| **Undergraduate** | Medical Student* or medical undergraduate* or medical school* or physician-trainee* or graduating trainee* or Medical Postgraduate*  MH "Students, Medical"  MH "Education, Medical, Undergraduate"  MH "Schools, Medical" |
| --- | --- |
| **Transition** | Transition* or progress* or shift* or trainee-to-trained |
| **Postgraduate** | medical graduate* or doctor* or Physician* or residen* or Newly qualified or new attending* or new* graduate* or clinician* or intern* or "Junior doctor*" or Registrar*  MH "Education, Medical, Graduate+"  MH "Physicians" |
| **Professional identity** | Professional identi* or Social Identi* or Occupational identi* or socialization  MH "Social Identification"  MH "Socialization" |

**Table 2. Condensed extraction table**

| Study ID | Authors | Year | Journal | Country | Study Design | Methods | Participants | Outcome | Key Findings | Study Design | T | Participant Type |
| --- | --- | --- | --- | --- | --- | --- | --- | --- | --- | --- | --- | --- |
| 11 | Satterfield & Becerra | 2010 | Med Educ | USA | Qualitative | Support group analysis / thematic analysis | Internal medicine residents | Stressors, coping, developmental challenges | Residents face longitudinal emotional/developmental challenges; peer support groups are critical; reframing and adaptation are key coping strategies | Qualitative (not longitudinal) | 31 | Both |
| 19 | Bearman et al. | 2011 | Adv Health Sci Educ | Australia | Qualitative | Interviews | New medical graduates | Participation in communities of practice; professional progression | Graduates transition through legitimate peripheral participation; belonging and contribution to teams shape identity | Qualitative (not longitudinal) | 32 | Postgraduate |
| 1 | Szymczak & Bosk | 2012 | J Health Soc Behav | USA | Qualitative | Ethnography (observation + interviews) | Internal medicine residents (3 programs) | Efficiency, time management, professional socialization | Efficiency as social norm and strategy; residents struggle with systemic barriers; workload exceeds available time | Qualitative (not longitudinal) | 41 | Postgraduate |
| 2 | De Lasson et al. | 2016 | BMC Med Educ | Denmark | Qualitative | Semi-structured interviews | Junior doctors (post-graduation) | Professional identity formation, group coaching | Group coaching supports PIF; peer support and reflection are key facilitators | Qualitative (not longitudinal) | 42 | Postgraduate |
| 21 | Jones et al. | 2016 | BMJ Open | UK | Qualitative (narrative) | Narrative interviews | Medical students and educators | Assistantship alignment; identity; readiness | Aligned assistantships support PIF and transition readiness; misalignment creates identity tension and unpreparedness | Quantitative longitudinal | 34 | Undergraduate |
| 12 | Wainwright et al. | 2017 | BMC Med Educ | UK | Qualitative | Interviews | Junior doctors accessing professional support | Distress, support needs, professional identity | Junior doctors in distress face identity threats; professional support units enable recovery and identity reconstruction | Qualitative longitudinal | 35 | Postgraduate |
| 9 | van den Broek W. et al. | 2017 | GMS J Med Educ | Netherlands | Qualitative/Mixed | Survey + qualitative | Final year medical students | Preparation for residency; transition readiness | Elective rotations support transition; students seek specialty exposure and confidence-building before residency | Qualitative longitudinal | 3 | Undergraduate |
| 7 | Monrouxe et al. | 2017 | BMJ Open | UK/Taiwan | Quantitative (longitudinal cohort) | Survey / validated scales | Junior doctors | Burnout, professional identity, workplace learning | Professional identity and team understanding are protective against burnout; gender and anxiety are significant factors | Mixed methods | 7 | Postgraduate |
| 14 | Ahmadinia et al. | 2019 | J Res Med Dent Sci | Iran | Qualitative | Interviews | Medical residents | Stressors affecting PIF during residency | Multiple stressors identified (workload, hierarchy, patient responsibility); social and institutional factors shape PIF | Qualitative (not longitudinal) | 44 | Postgraduate |
| 18 | Yardley et al. | 2020 | Adv Health Sci Educ | UK | Qualitative (narrative analysis) | Narrative interviews | Junior doctors / recent graduates | Identity and responsibility at transition | Transition involves rapid shift in identity and accountability; responsibility triggers identity reconstruction; relational context is crucial | Qualitative (not longitudinal) | 33 | Postgraduate |
| 10 | Brown J et al. | 2020 | Med Educ | UK | Qualitative (case study) | Audio-recordings + interviews (critical realism) | GP trainee/supervisor pairs | Identity formation within supervisory relationship | Supervisory relationship is key to PIF; identity co-constructed through interaction; 'cultural worlds' frame useful | Qualitative longitudinal | 39 | Postgraduate |
| 8 | Chang et al. | 2020 | Academic Medicine | USA | Qualitative | Semi-structured interviews (grounded theory) | PGY-1 residents (n=10) | Identity, professional growth, transition experience | PIF is core construct of transition; residents navigate responsibility, relationships, and self-concept | Qualitative longitudinal | 36 | Postgraduate |
| 17 | van den Broek et al. | 2020 | Teach Learn Med | Netherlands | Quantitative/Mixed | Survey (Social Identity Theory framework) | Medical students and new practitioners | Social identification with profession; PIF | Social identification strengthens across transition; group membership and belonging central to PIF | Survey / Quantitative longitudinal | 45 | Both |
| 13 | Montagna et al. | 2021 | BMJ Open | Brazil/Ireland | Qualitative | Semi-structured interviews | Young doctors transitioning during COVID-19 | Transition experience, identity, adaptation to crisis | Pandemic intensified transition challenges; accelerated identity shifts; need for peer support and institutional guidance | Qualitative (not longitudinal) | 27 | Postgraduate |
| 4 | Chaou et al. | 2021 | BMC Med Educ | Taiwan | Longitudinal mixed-methods | Surveys + qualitative data | Medical students transitioning to postgraduate | Preparedness for clinical practice | Preparedness evolves over time; clinical exposure and feedback are key determinants | Qualitative longitudinal | 29 | Postgraduate |
| 6 | Brown MEL et al. | 2021 | Adv Health Sci Educ | UK | Qualitative (phenomenological) | Participant-voiced poetry / narrative | New doctors (recently qualified) | Lived experience of transition; identity | Transition involves emotional upheaval; sense of responsibility, isolation and belonging; identity reconstruction | Qualitative longitudinal | 30 | Both |
| 15 | Wolff et al. | 2021 | Med Educ Online | USA | Mixed-methods / intervention study | Coaching program evaluation | Medical students and new residents | Coaching intervention; transition support | Facilitated coaching improves transition; emotional and professional preparedness enhanced; peer mentoring beneficial | Qualitative longitudinal | 40 | Postgraduate |
| 16 | Santivasi et al. | 2022 | Perspect Med Educ | USA | Qualitative (grounded theory) | Semi-structured interviews | Internal medicine residents (n=23) | Identity tensions, reframing, PIF | Residents navigate tensions between ideals and reality; reframing is adaptive strategy for PIF; context enables and constrains identity | Qualitative (not longitudinal) | 43 | Postgraduate |
| 3 | van Duin et al. | 2022 | Med Educ | Netherlands/Multi-country | Qualitative | Interviews | Junior doctors | Interprofessional collaboration, team learning | IPL occurs implicitly; junior doctors navigate complex professional landscapes without structured guidance | Qualitative longitudinal | 28 | Postgraduate |
| 5 | Zink et al. | 2022 | AEM Educ Train | USA | Qualitative | Written reflections / qualitative analysis | Emergency medicine interns (PGY-1) | Emotional experience, hopes, fears at transition | Interns express excitement and anxiety; fear of inadequacy and patient harm; need for support and belonging | Qualitative (not longitudinal) | 24 | Postgraduate |
| 20 | Homberg et al. | 2023 | Med Teach | Germany | Quantitative (content analysis) | Self-reports / content analysis | Final year medical students | Experience-based learning; preparedness | Clinical experiences in final year vary widely; self-reports reveal gaps in preparedness; structured exposure needed | Qualitative longitudinal | 38 | Undergraduate |
